# A colliding maxillary sinus cancer of adenosquamous carcinoma and small cell neuroendocrine carcinoma - a case report with *EGFR *copy number analysis

**DOI:** 10.1186/1477-7819-8-92

**Published:** 2010-10-20

**Authors:** Shiang-Fu Huang, Wen-Yu Chuang, Sou-De Cheng, Li-Jen Hsin, Li-Yu Lee, Huang-Kai Kao

**Affiliations:** 1 Department of Otolaryngology, Chang Gung Memorial Hospital and Chang Gung University, Taiwan; 2 Department of Pathology, Chang Gung Memorial Hospital and Chang Gung University, Taiwan; 3 Department of Anatomy, Chang Gung University, Tao-Yuan, Taiwan; 4 Department of Plastic Surgery, Chang Gung Memorial Hospital and Chang Gung University, Taiwan

## Abstract

**Background:**

Small cell neuroendocrine carcinoma (SNEC) of maxillary sinus is a rare and aggressive malignancy. A tumor with squamous cell carcinoma, adenocarcinoma and SNEC co-existence is extremely rare.

**Case presentation:**

We present a colliding tumor of squamous cell, adenocarcinoma and SNEC in maxillary sinus. The clinical features, diagnosis and EGFR flourescence in situ hybridization (FISH) study are presented. A 52-year-old female had a 1-month history of progressing left cheek swelling and purulent rhinorrhea. Magnetic resonance imaging showed a tumor involving left maxilla and orbital floor. Excision of tumor was done and the defect was reconstructed with free flap. The pathology revealed a malignant tumor composed of squamous cell carcinoma, adenocarcinoma and SNEC components. EGFR FISH study showed no gene amplification in 3 components of this tumor. The tumor progressed rapidly and the patient expired at 8 months after surgery.

**Conclusion:**

A colliding tumor of squamous cell, adenocarcinoma and neuroendocrine carcinoma in maxillary sinus was aggressive in behavior and the treatment response was poor due to the complexity of tumor.

## Introduction

Carcinoma of the paranasal sinuses accounts for about 0.3% of all cancers [1]. Most of the malignancies in paranasal sinus is squamous cell carcinoma (SCC) and followed by adenocarcinoma. Small cell neuroendocrine carcinoma (SNEC) is a rare tumor in head and neck region and it occurs most frequently in larynx [2]. Paranasal sinuses are uncommon primary sites for the occurrence of extrapulmonary SNECs. Only small series and case reports were available to date for primary sinonasal tract SNEC [3]. Foci of squamous or glandular differentiation in SNECs were occasionally noted [4,5]. The collision of three components (squamous cell, adenocarcinoma and neuroendocrine cells) in a solid tumor was very rare. Only two reports of combined adenosquamous and large-cell neuroendocrine carcinoma were reported in the literature [6,7].

Despite extensive treatment, SNECs in head and neck had poor prognosis and high rates of recurrence and distant metastasis [3]. Epidermal growth factor receptor (EGFR) antagonists and monoclonal antibodies were found to have promising results in non-small lung cancer and colon cancer [8,9]. In head and neck SCC, several EGFR inhibitors have been studied alone or in combination with cisplatin/carboplatin and were found to have modest response rates [10,11]. The target therapies provide new options to traditional therapies. In a tumor of three different histologies (squamous cell, adenocarcinoma and neuroendocrine cells) and aggressive in behavior, we investigated the EGFR copy number by fluorescence in situ hybridization (FISH) in each component of the tumor. The feasibility of EGFR target therapy in such a malignant tumor was discussed in the text.

### Case Report

**Figure 1:**
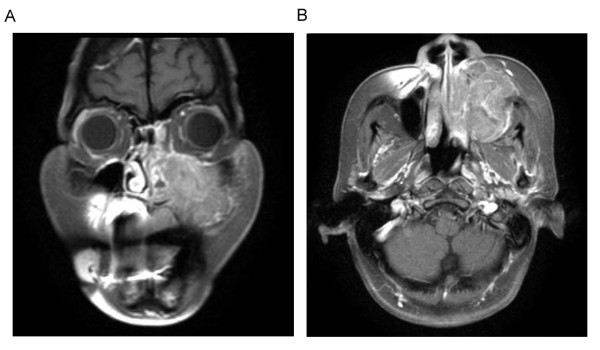
**Post-gadolinium contrast-enhanced T1-weighted coronal magnetic resonance imaging (MRI), with fat saturation, showed an enhancing and destructive mass with indistinct borders in left maxillary sinus**. (A) Coronal view. (B) Axial view.

**Figure 2:**
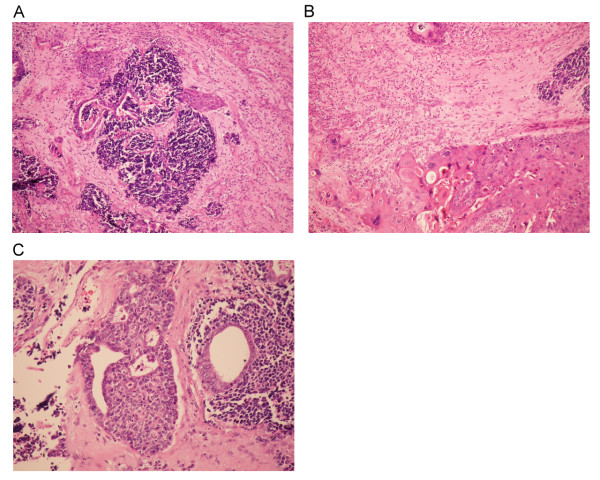
**A. Histologic appearance of combinations of small, ductal and squamous cellular components of the tumor**. (H & E, x100) B. Transitional area between squamous cell carcinoma and neuroendocrine cells. (H & E, x200) C. Transitional region between glandular and neuroendocrine components. (H & E, x200).

A 52-year-old female with history of hypertension and type II diabetes mellitus, came to our clinic with the chief complaint of left cheek swelling and persistent purulent mucoid nasal discharge from left nostril for one month. Physical examination revealed a nasal tumor and a bulging mass in hard palate. Biopsy under sinoscope was done and the pathology showed a SNEC with positive neuron-specific enolase, CD 56, synaptophysin, Cam 5.2 and focally AE1:AE3 from immunohistochemistry. Computed tomography (CT) scan and MRI (Figure 1) revealed a destructive lesion involving all walls of left maxillary sinus. There was no evidence of distant metastasis from bone scan and abdominal sonography. Left total maxillectomy was performed with free flap reconstruction (left anterior lateral thigh flap) following the resection. Microscopically, bony invasion was evident and the final pathology revealed a malignant tumor composed of SNEC, SCC and adenocarcinoma (Figure 2). Adjuvant chemotherapy with FT-207, leukovorin, and cisplatin were given. Regional recurrence at bilateral neck lymph nodes occurred at 2 months and lung metastasis at 6 months after surgery. The patient expired later due to sepsis and had an overall survival of 8 months after diagnosis.

### FISH Assay and Analysis

EGFR copy numbers were investigated by FISH using the LSI *EGFR *SpectrumOrange/CEP 7 SpectrumGreen probe (Vysis; Abbott Laboratories, Downers Grove, IL) and this was carried out using the manufacturer's protocols. In brief, section slides were incubated at 56°C overnight, deparaffined, dehydrated, and treated with 0.2 N HCl (pH 2.5) for 20 min. This was followed by 1 M sodium thiocyanate (Sigma-Aldrich Corp., St. Louis, MO) in 1 M Tris (pH 8.0) at 82°C for 20 min, and then the specimens were digested with 0.4% pepsin (Sigma-Aldrich Corp., St. Louis, MO) in 0.9% NaCl (pH 2.35) for 15 min. The samples were briefly rinsed with ddH_2_O and 2 × SSC between steps. After fixation in 4% formaldehyde for 5 min, each slide had the probe set applied to the selected area, and the hybridization area was covered with a plastic coverslip and sealed with a glue gun before heating at 75°C for 10 min in an OmniGene system (Hybaid Ltd., Middlesex, United Kingdom) to allow co-denaturation of the chromosomal and probe DNAs. Hybridization was then carried out in a humidified oven at 37°C for 18 hours, and this was followed by postwashing in 0.3% Nonidet P40 (BDH, England) in 2 × SSC at 55°C for 4 min, in 2 × SSC at 55°C for 5 min, and finally twice in 2 × SSC at room temperature for 5 min. After being counterstained with DAPI for 5 min, the slides were mounted with Vectashield mounting medium (Vector Laboratories, Burlingame, CA), and scored under an epifluorescence microscope using a Plan Neofluar 100 × objective (Axiophot, Zeiss, Germany) with FITC/TRITC dual and DAPI/FITC/TRITC riple pass filters (Chroma Technology Corp., Bellows Falls, VT).

**Figure 3:**
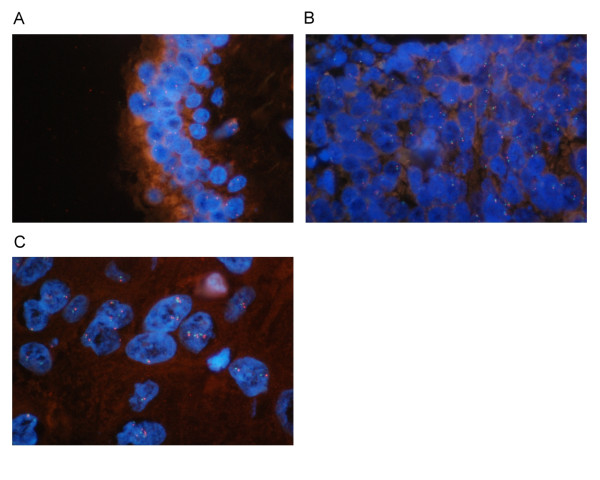
***EGFR *gene FISH analysis. The field was observed by the triple band filter (1000×)**. (A) Mostly monosomy in ductal cells. (B) Both disomy and monosomy in neuroendocrine cells. (C) Some cells with increased *EGFR *copy number in squamous cell components.

The EGFR copy number was mostly monosomy in the ductal component, disomy in the neuroendocrine component and some trisomy in the squamous cell component (Figure 3). No EGFR amplification was found in the FISH analysis.

## Discussion

Most of maxillary sinus cancer was SCC and adenocarcinoma. SNEC of the sinonasal region is the least common of the sinonasal carcinomas with neuroendocrine differentiation. The SCC or adenocarcinoma in maxillary sinus developed from the lining mucosal epithelium or metaplasia. The histogenesis of the neuroendocrine differentiated tumors is not clear. Whether those cells stem from a common pluripotent precursor or from metaplastic neuroendocrine cells is still unclear. It has been postulated that, outside the lung, this tumor is derived from the neuroendocrine APUD cells, which are widely distributed in the body. The tumors are composed of small-sized regular cells arranged in broad sheets, nests, and cords. Many of the cells contain cribriform nuclei with a fine reticular chromatin pattern and small to moderate amounts of cytoplasm [12]. Immunohistochemical study is essential to make an adequate differential diagnosis from other malignant tumors such as lymphoma, rhabdomyosarcoma, undifferentiated nasopharyngeal carcinoma, and undifferentiated sinonasal carcinoma. SNEC has been reported to stain strongly with synaptophysin and CD56 nerve cell adhesion molecule and weakly with chromogranin A and CAM 5.2/AE-1 [13].

Yazawa et al. had investigated the clonality of colliding tumor which composed of adenocarcinoma, SCC and large cell neuroendocrine carcinoma [7]. The clonality results were similar in adenomatous and squamous components and different in neuroendocrine component. There were some limitations in that study due to possible DNA fragmentations in formalin fixed tissues. However, it confirmed the adenomatous and squamous components were the same origin and classified it as a colliding tumor of adenosquamous carcinoma (ASC) and neuroendocrine carcinoma. In the literature, ASC is an unusual neoplasm and often misdiagnosed [14]. In our patient, the colliding tumor could originate from a mixture of ASC and the SNEC which gives the tumor a feature of 3 different histologies.

The treatment of SNECs varied considerably over time which includes surgery followed by radiotherapy, concurrent chemo-radiotherapy with or without surgery, and chemotherapy with cisplatin and eoposide followed by radiation or surgery [15]. No consensus yet exists about its treatment. The management of maxillary sinus ASC is also controversial due to its rarity. The prognosis in cases of head and neck SNEC and ASC is very poor because of the high metastatic rate observed. [3,14] In our patient, the tumor recurred and metastasized rapidly after surgery. Its response to chemotherapy was poor. The diverse components of the tumor could possibly account for its non-responsiveness to chemotherapy and aggressive behaviors.

Recently, EGFR targeting agents such as Cetuximab (C225, Erbitux™), a human-murine chimeric monoclonal antibody, gefitinib and erlotinib were developed. They are less toxic in side effects than cheomotherapies. In locoregionally advanced head and neck SCC, concomitant high-dose radiotherapy plus Cetuximab had been shown to improve locoregional control and reduces mortality [16]. In chemotherapy refractory head and neck cancers, some are responsive to EGFR target treatment. However, from EGFR copy number analysis in each component of the tumor, no EGFR amplification was found. EGFR targeting agents might have limited roles in this rare tumor.

## Conclusion

A colliding malignant tumor of SCC, adenocarcinoma and SNEC is rare in head and neck malignancies. The diagnosis of this tumor usually could be made after excision of tumor. Both adenosquamous carcinoma and SNEC have aggressive behaviors. The colliding tumor of these components also carries a poor prognosis. EGFR copy numbers in all three histologies were not increased and the efficacy of anti-EGFR target therapy could be limited in this rare malignancy.

## Consent

Written informed consent was obtained from the patient for publication of this case report and accompanying images. A copy of the written consent is available for review by the Editor-in-Chief of this journal.

## Competing interests

The authors declare that they have no competing interests.

## Authors' contributions

HSF and HLJ conceived the idea for the manuscript, conducted a literature search, and drafted the manuscript. HSF performed surgery, obtained images and critically write the manuscript. CWY and LLY provided and reviewed pathological images. CSD performed flourescence in-situ hybridization study. HLJ and KHK critically revised the manuscript. All authors read and approved the final manuscript.
